# An Invisible Salient Landmark Approach to Locating Pedestrians for Predesigned Business Card Route of Pedestrian Navigation

**DOI:** 10.3390/s18093164

**Published:** 2018-09-19

**Authors:** Zhixiang Fang, Yuxin Jiang, Hong Xu, Shih-Lung Shaw, Ling Li, Xuexian Geng

**Affiliations:** 1 State Key Laboratory of Information Engineering in Surveying, Mapping and Remote Sensing, Wuhan University, 129 Luoyu Road, Wuhan 430079, China; zxfang@whu.edu.cn (Z.F.); yxjiang@whu.edu.cn (Y.J.); whu_liling@foxmail.com (L.L.); 2 School of Urban Construction, Wuhan University of Science and Technology, Wuhan 430065, China; 3 Department of Geography, The University of Tennessee, Knoxville, TN 37996-0925, USA; sshaw@utk.edu; 4 Guangzhou Institute of Geography, Guangzhou 510070, China; 5 Electronic Information School, Wuhan University, Wuchang District, Wuhan 430072, China; gxx@whu.edu.cn

**Keywords:** landmark, pedestrian route, sensor signal, route guidance, smartphone navigation, Dempster-Shafer theory of evidence

## Abstract

Visual landmarks are important navigational aids for research into and design of applications for last mile pedestrian navigation, e.g., business card route of pedestrian navigation. The business card route is a route between a fixed origin (e.g., campus entrance) to a fixed destination (e.g., office). The changing characteristics and combinations of various sensors’ data in smartphones or navigation devices can be viewed as invisible salient landmarks for business card route of pedestrian navigation. However, the advantages of these invisible landmarks have not been fully utilized, despite the prevalence of GPS and digital maps. This paper presents an improvement to the Dempster–Shafer theory of evidence to find invisible landmarks along predesigned pedestrian routes, which can guide pedestrians by locating them without using digital maps. This approach is suitable for use as a “business card” route for newcomers to find their last mile destinations smoothly by following precollected sensor data along a target route. Experiments in real pedestrian navigation environments show that our proposed approach can sense the location of pedestrians automatically, both indoors and outdoors, and has smaller positioning errors than purely GPS and Wi-Fi positioning approaches in the study area. Consequently, the proposed methodology is appropriate to guide pedestrians to unfamiliar destinations, such as a room in a building or an exit from a park, with little dependency on geographical information.

## 1. Introduction

Computer-assisted pedestrian navigation is an area that requires ongoing research because of people’s varying abilities [1], the complexity of various environments, the localization problem in indoor and outdoor environments [2,3,4,5,6], and data modeling [7,8] for navigation applications. Current researchers and industrial practitioners usually focus on the theory and technology of locating and guiding pedestrians and often provide shortest-path services. Plentiful localization techniques contribute the coordinates (x, y, z) of navigation devices that are commonly used to guide pedestrians location by location through the use of digital navigation maps [9], street view or scene [10], or visual landmarks [11,12]. Current localization techniques and their corresponding pedestrian navigation applications are fully constrained by usability of signals of opportunity [2] in the environment, for example, absence of Wi-Fi access points (APs), shielding of Global Navigation Satellite Systems (GNSS), and visibility of landmarks. Typically, however, only a few sensors in smartphones or navigation devices are used to contribute to localization or navigation, such as Wi-Fi-based positioning [13,14] and its Kalman filter-based error smoothing [15,16], Bluetooth inquires [17], GPS, accelerometers [18], and drift compensation algorithm with invisible landmarks [19]. Other signals from built-in sensors in smartphones or navigation devices are not yet fully utilized in current navigation applications, for instance the gyroscope, gravity, orientation, light, proximity and so on. Each sensor has characteristic changes to its signals during the process of pedestrian navigation as a result of the influence of terrain and pedestrian behavior [1,18,20]. The means of determining characteristic changes in the signals from built-in sensors in smartphones or navigation devices, and using them as “invisible salient landmarks” to guide pedestrians has not been addressed in the literature. These invisible landmarks could be very helpful to understand the real-time locations and movements of pedestrians for smart navigation applications.

This paper introduces an invisible landmark-based approach to locate pedestrians in indoor and outdoor environments, that is, an improved Dempster-Shafer (D-S) theory of evidence, which is proposed to locate pedestrian by considering co-existing phenomena in the form of sensors’ signal change characteristics. All signals from the sensors are sequenced in a linear reference manner along a target “business card” route. The target route is requested by the proposer from a specific location (i.e., entrance of university campus) to a particular destination of him, such as the office. Then, the changing characteristics of various sensors and their combinations are analyzed to build up the frame of discernment in the theory of evidence. In this paper, we propose:

(1) An improved D-S theory of evidence is introduced by integrating the co-existing phenomena of sensors’ signal change characteristics. This approach is distinguished from navigation methods that use fingerprint-based localization and navigation applications by its focus on the nature of the combined rather than individual signal changes.
(2) The similarity of real-time data of co-existing sensors to the predefined evidence framework is defined to refine the basic belief assignment in the theory of evidence.
(3) A match error-based sensor weight assignment approach is proposed to handle conflicts of evidence processing.

Thus, the invisible landmark-based pedestrian locating approach based on the D-S theory of evidence is a useful guide to allow newcomers to follow predesigned business card routes.

The remainder of this paper is structured as follows: Section 2 outlines previous research, while Section 3 describes the sensors in smartphones and their signals, introduces a framework of the proposed approach, and then describes an improved approach of D-S theory for pedestrian locating. Section 4 describes the experiments and results and Section 5 states some conclusions and the direction of future works.

## 2. Related Work

### 2.1. Landmark-Based Navigation

Landmark-based navigation tasks are broadly discussed in the disciplines of cognition, neuroscience, geographical information science (GIS), computing, and communication.

There is rich literature in the disciplines of cognition and neuroscience on the spatial cognitive aspects of landmark-based instruction. Researchers have studied the perceptual, cognitive, and contextual aspects of landmarks in wayfinding; for example, landmark-based instructions enable pedestrians to comprehend efficiently the visual prominence, semantic salience, and structural significance of landmarks [7,21,22,23,24]. These studies focus on spatial behavioral factors of people, for instance, spatial abilities, spatial cognition, and spatial decision-making [25]. Some other research works have been published that investigated the landmark-based spatial cognition of different areas of the brain [26], neural activity [27,28], environmental psychology [29], and cognitive map and decision-making recognition processes [30].

In the discipline of GIS, many studies focus on the collection of landmark information, the use of landmarks in route instructions, and the modeling of landmarks in GIS: for example, discovering landmark preferences from photo postings [31], extracting landmarks from laser scanning [32], modeling landmarks using scene graphs [33], including landmarks in routing Instructions [11], landmark location in localization system for urban areas [34], developing landmark-based pedestrian navigation models [7] and three-dimensional navigable data models [35], and estimating cartographic communication performance [36]. Furthermore, some GIS-based pedestrian navigation services or systems have been developed for personal guidance for indoor and outdoor environments [37,38,39,40].

In the disciplines of computing and communication, many studies have been conducted on scene landmark recognition, understanding, and classifications for applications, including robot navigation. For example, identifying landmarks in urban scenes [41], vision-based indoor scene analysis for detecting natural landmarks [42], assessing the recognition time in real scenes [43], projecting dynamic images for scene understanding [44], classifying landmarks in large-scale image collection [45], visualizing landmarks to support spatial orientation [46], and even developing an image-based indoor navigation system [47].

### 2.2. Sensor-Based Pedestrian Localization

Much research work has focused on the usage of inertial sensors in smartphones or navigation devices for pedestrian localization, for example: GPS, infrared [48], Ultra Wide Band (UWB) or ultrasound [49,50], laser [51], Wireless Local Area Net (WLAN), Wi-Fi [52] or Li-Fi [53], radio-frequency identification (RFID) [54], Bluetooth [55], wireless sensor network [56], light-emitting diode (LED) [57], Global System for Mobile Communications (GSM) [58], and other inertial sensor-based localization [59,60,61]. The general positioning approaches are based on the theory of fingerprinting or triangulation, for instance, to overcome the effects of multipathing and shadowing [62] in the positioning environment. A recent publication [4] proposed a turbo received signal strength (RSS) model-based indoor algorithm for crowded scenarios. The main perspective of these sensor-based pedestrian localization research works was feature analysis of sensor signals related to digital geographical maps, fingerprinting [4], or activities [18]. In these studies, several sensors or information fusion-based approaches of using several signals from sensors were used to contribute to pedestrian localization. A recent, robust crowdsourcing-based indoor localization system that makes full use of signals collected by smartphones from multiple sensors for pedestrian localization was presented [3]. It adopts a novel and promising perspective to solve the pedestrian localization problem. Our study was inspired by this approach and tries to make full use of signal features that are highly correlated with predefined pedestrian navigation routes to ensure accurate route guidance in both indoor and outdoor environments.

## 3. Materials and Methods

### 3.1. Sensors Signals in Smartphones

Nowadays, the functionality of smartphones far exceeds simple communication. Current expectations are such that phones have applications for entertainment, practice, and learning. Thus, smartphones integrate more and more sensors (e.g., GPS, Wi-Fi, light meter, magnetometer, gyroscope, and accelerometer) to meet people’s expectation for technological assistance in day-to-day existence.

The sensors in smart phones can be classified as three categories: motion, environmental, and position sensors. A gyroscope, as a motion sensor, can effectively detect data changes caused by pedestrian movement. The light sensor is an environmental sensor, which can measure light intensity in the pedestrian’s environment. GPS, Wi-Fi and magnetometer sensors are position sensors although it is influenced by the Sun. GPS sensors can determine a person’s position both indoors and outdoors. Wi-Fi can be used to triangulate a pedestrian’s location and provide internet services for them. This paper will use the gyroscope, light sensor, GPS, Wi-Fi, and magnetometer sensors to match the locations of pedestrians with predetermined routes that connect origins and destinations, and recode all the signals of these sensors while traveling along the route.

### 3.2. Framework of the Proposed Approach

This paper proposes an invisible landmark-based navigation guidance approach, based on the D-S theory of evidence, which aims to guide pedestrians to their destination smoothly by following business card route. The business card route is represented by a sequence of signals detected by the smartphone’s sensors between the origin and the destination of this route. The proposed approach compares the real-time signals in smartphones with the precollected signals of the business card route so as to sense the current location of pedestrian.

**Figure 1:**
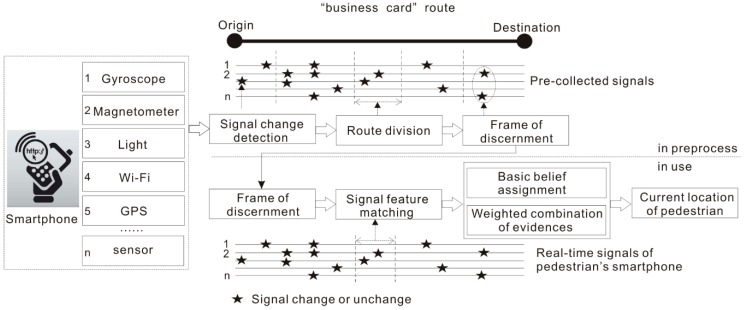
Framework of the proposed approach.

Figure 1 illustrates the framework of the proposed approach. This approach requires the initial collection of all signals of smartphone’s sensors along the whole business card route. These precollected signals are used to build up the frame of discernment in the theory of evidence by applying two important steps. The first step is to detect signal change characteristics, including significant changes and unchanged patterns. The second step is to divide the routes into route segments according to the changes of the signals. The combination of signal changes forms the frame of discernment, which provides fundamental evidence to the process of signal feature matching while pedestrians navigate, dependent only on their smartphones. When a pedestrian follows the predefined business card route, they need to check their locations with the help of a navigation app on their smartphones. This app will compare the real-time signal of the smartphones with the frame of discernment by evidence checking according to the combination rule of evidence for each route segment. The determination is closely dependent on the basic (belief) probability assignment (BPA) [63] and the proposed weighted combination of evidence in the theory of evidence.

### 3.3. Pedestrian Location Matching Algorithm Based on an Improved Dempster-Shafer Theory of Evidence

This section introduces the basic concept and approach of the D-S theory of evidence, and then proposes improvements for determining the location of pedestrians.

#### 3.3.1. The D-S Theory of Evidence

The D-S theory of evidence, also known as the theory of belief functions, is an efficient Bayesian framework for approximate reasoning and decision-making in uncertain environments, which was originally proposed by Dempster [63] and improved by Shafer [64]. There are three key components of the theory: defining the frame of discernment, obtaining the basic belief for one question from subjective probabilities for a related question [64], and Dempster’s rule for combining such degrees of belief [63].

Let $\theta =\{\theta_{1},\theta_{2}\ldots ,\theta_{n}\}$ denote the set of all possible situations for a given event, which is called the frame of discernment [64]. Each element of this set is independent of the others, i.e., $\theta_{i}\cap \theta_{j}=\phi ,\forall i,j=\{1,\ldots ,n\}$. The frame of discernment in this paper is derived from smartphone sensor data. The D-S theory of evidence assigns a belief mass to each element as a basic probability assignment function $m:2^{\theta }\to [0,1]$, which satisfies the following conditions: the mass of the empty set m(ϕ)=0 and $\sum_{A\in \theta }m\left(A\right)=1$. The function *m* is a BPA on θ. Then, if A is a subset of θ, m(A) is a function which can be interpreted as the degree of belief that that the set *A* belongs to the set θ, which satisfies 0≤m(A)≤1,∀A∈θ&A≠ϕ. In practice, Shafer’s framework can use an interval [Bel(A),Pl(A)] to represent the belief about propositions. The interval is bounded by two values of the belief function (Bel) and the plausibility function (Pl) [64], where Bel(A) measures the total belief that the object is in A, and Pl(A) measures the total belief that can move into A. The two functions are defined as follows:

(1) $$Bel\left(A\right)=\sum_{B\in A}m\left(B\right)$$

and

(2) $$Pl\left(A\right)=\sum_{B\cap A=\phi }m\left(B\right)$$

Therefore, the interval represents the level of uncertainty based on the evidence in the framework. For example, the interval [0,0] implies that it is completely unsupported; whereas, the interval [1,1] indicates full support.

In the case of multiple sources of evidence, the BPAs could be combined to yield a new BPA function M, which is defined as follows according to Dempster’s combination rule:

(3) $$M\left(A\right)=\left\{ \begin{array}{ll} \frac{1}{K}\sum_{A_{1}\cap A_{2}\cap \cdots \cap A_{n}=A}m_{1}\left(A_{1}\right)\cdot m_{2}\left(A_{2}\right)\cdots m_{n}\left(A_{n}\right) & A\neq \phi \\ 0 & A=\phi \end{array}\right.$$

$$K=\sum_{A_{1}\cap \cdots \cap A_{n}\neq \phi }m_{1}\left(A_{1}\right)\cdot m_{2}\left(A_{2}\right)\cdots m_{n}\left(A_{n}\right)=1-\sum_{A_{1}\cap \cdots \cap A_{n}=\phi }m_{1}\left(A_{1}\right)\cdot m_{2}\left(A_{2}\right)\cdots m_{n}\left(A_{n}\right)$$

where K is a normalization constant, $m_{i}$ is the BPA of evidence i, and $A_{i}$ is the subset of evidence i.

Two main factors can lead to conflicts of evidence in the theory of evidence, namely: sensor unreliability, caused by equipment failure or limitations, and incomplete knowledge of the world [65]. As long as discernment distributions are not completely consistent, conflicts of evidence will occur during evidence fusion and have a great influence on the accuracy of the result of evidence combination. Two main methods are used to solve this evidence conflict. One is improving the combination rule, which starts from the original flaws of the evidence synthesis. The most representative method proposed by Yager [66] assumes that conflicting evidence also carries some useful information that can be assigned to unknown items and effectively used. The other is the modification of the sources of evidence in order to avoid a conflict of evidence [67].

#### 3.3.2. Improved Approaches of D-S Theory for Pedestrian Location

This paper improves the D-S theory by combining the accompanying rules of evidence and their processing of conflicts of evidence. In the implementation of D-S, this paper generates evidence frameworks for individual route segments. Each segment has some associated evidence from sensors. The following subsection will firstly introduce the path division, and then introduce the consequential improvement in D-S theory.

##### 3.3.2.1. The Evidence Framework Based on Sensor Changes in Target Routes

Before establishing the frame of discernment, the user conducts several complete walks along the target business card route, and collects the sensor data of the smartphone on this route using any sensor data acquisition application. For each sensor, this study divides the route into segments according to the changes in its data. Consequently, this study could obtain the route segment set for each kind of sensor, namely, $L_{Magx}$, $L_{Magy}$, $L_{Gyro}$, $L_{Light}$, $L_{Wi-Fi}$, and $L_{Lon\&Lat}$, which are used to establish the evidence framework based on divided route segments. Each route segment must have at least one sensor change feature. The combination of change features within a route segment build up the evidence framework for each route segment.

**Figure 2:**
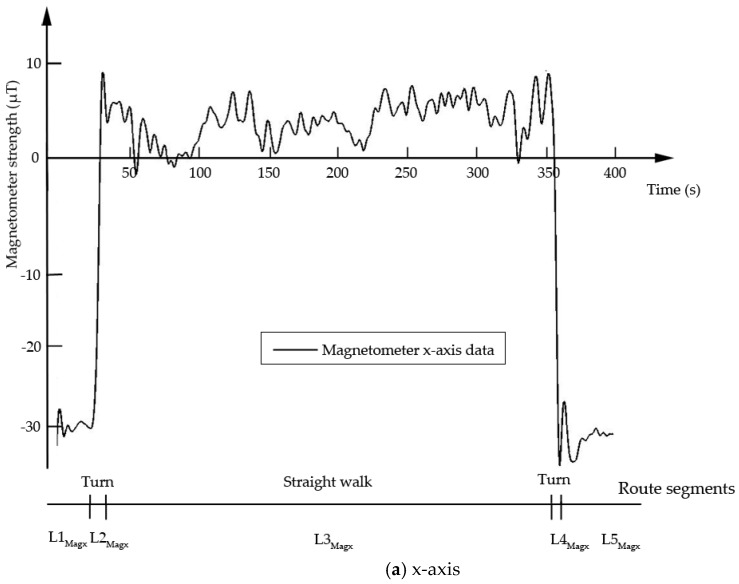

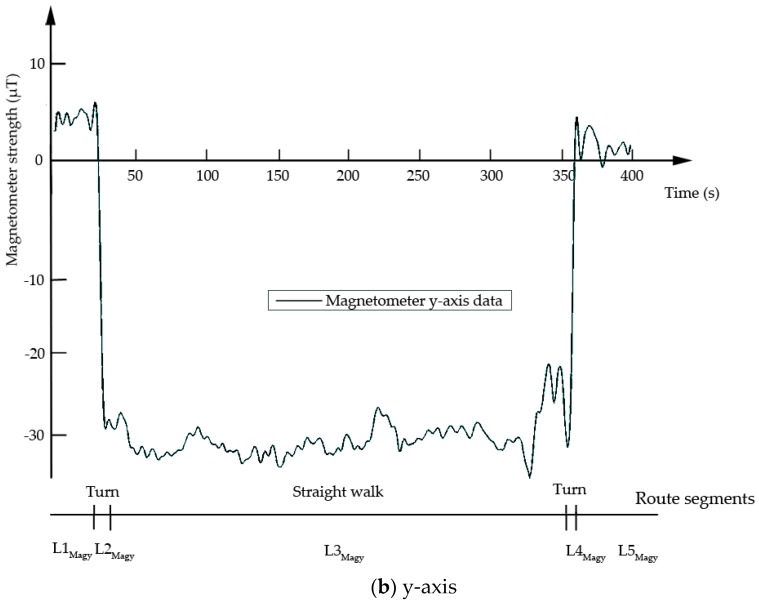
Magnetometer data resolved along the x-axis (**a**) and y-axis (**b**).

The first kind of sensor is a magnetometer, which is an instrument that measures the direction and strength of a magnetic field at a particular location. For a fixed point, the geomagnetic field can be decomposed into two horizontal components and a component that is perpendicular to the ground (x,y,z). The vector sum of the two horizontal components points to magnetic north, so the geomagnetism in the current environment can be identified only using the magnetometer x- and y-component data. In some locations, magnetometer readings are stable, but the magnetic values change when the environment is changing. Specifically, it may exhibit a sudden increase or decrease in value, and the variation in change quantity can reach 20 μT or more. The character of both the changes and lack thereof in the environment can be used as evidence of discernment. For example, Figure 2 illustrates two turns and the associated significant changes in magnetometer data. Consequently, this route can be divided into five segments based on the change features exhibited by the magnetometer, which indicate turns along the route. Based on the above division, this study could generate route segment sets $L_{Magx}$ and $L_{Magy}$ according to the x- and y-component data of magnetometer individually. The route segment set $L_{Magx}=\{L1_{magx},\;L2_{magx},\;L3_{magx},\;L4_{magx},\;L5_{magx}\}$ in Figure 2a, and $L_{Magy}=\{L1_{magy},\;L2_{magy},\;L3_{magy},\;L4_{magy},\;L5_{magy}\}$ in Figure 2b.

**Figure 3:**
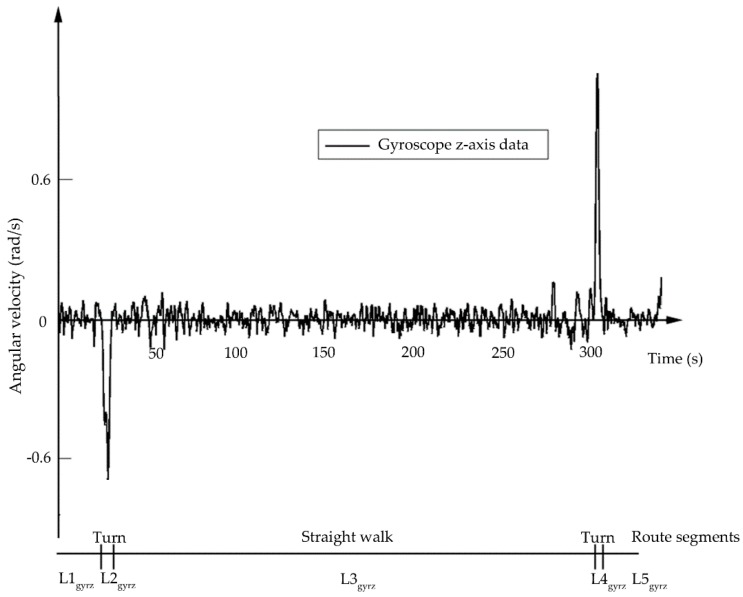
Gyroscope data.

The second kind of sensor is the gyroscope. This sensor is able to detect the angular velocity of a moving object, which allows the recognition of turning. In the process of turning, the absolute value of angular velocity obtained by the gyroscope will increase significantly. However, in the situation of smooth walking, the z-axis value of the gyroscope is nearly zero. The positions of left and right turns correspond to the peaks and troughs of the gyroscope data, respectively, as shown in Figure 3. Any simple moving window algorithm can detect the peaks and troughs in the data. The found peaks and troughs can be used to divide the target route into segments for constructing the evidence framework, such as the route segment set $L_{gyro}=\{L1_{gyro},L2_{gyro},L3_{gyro},L4_{gyro},L5_{gyro}\}$ in Figure 3.

**Figure 4:**
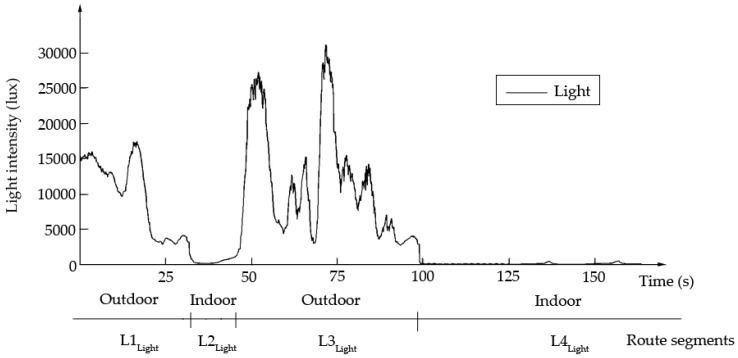
Light data.

The third kind of sensor is the light meter. Figure 4 illustrates an example of light intensity data within variable and stable environments, which can be distinguished by the first differential of light intensities, and indicates the divided segments by light intensity. Five thresholds {{−250,250}, {−200,200}, {−150,150}, {−100,100}, {−50,50}} were trialed as the first difference in light intensities. The experiment showed that a threshold of {−100,100} resulted in segmentation that was close to reality. Each segment had a unique light characteristic, either of fluctuation or stability. The route segment set $L_{light}=\{L1_{light},\;L2_{light},\;L3_{light},\;L4_{light}\}$ in Figure 4.

**Figure 5:**
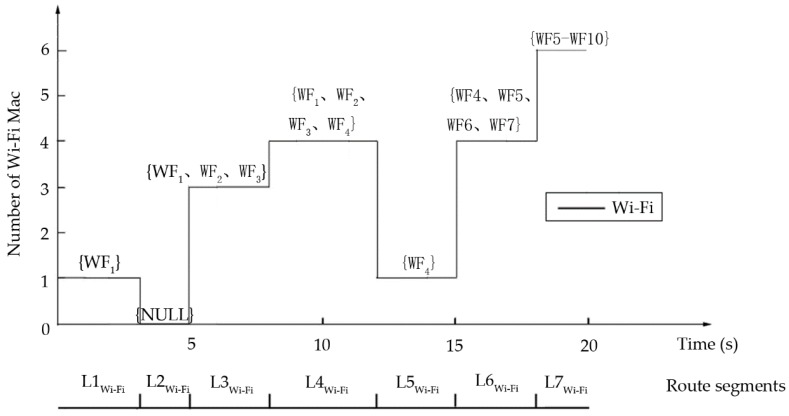
Wi-Fi Media Access Control (MAC) data.

**Figure 6:**
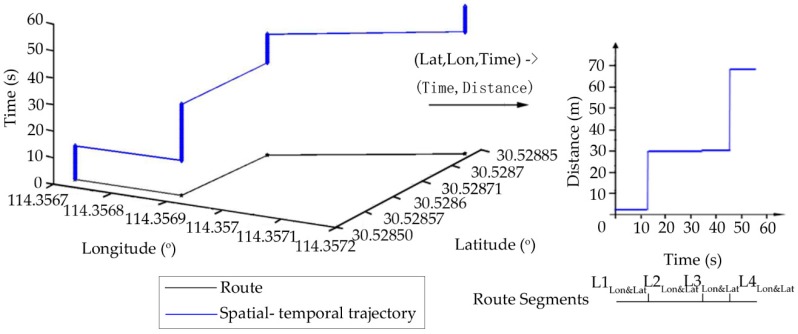
Data of Global Positioning System.

The other sensors are for Wi-Fi and GPS and the processes associated with them are very similar. The Wi-Fi signal distribution has a certain range, and the mobile phone can accept that signal anywhere within that range. Therefore, Wi-Fi Media Access Control (MAC) address along the target route could be used to divide it into segments. Each segment has a limited Wi-Fi MAC address set and the neighbor segments will have a different Wi-Fi MAC address. For example, the route segment set $L_{Wi-Fi}=\{L1_{Wi-Fi},\;L2_{Wi-Fi},\;L3_{Wi-Fi},\;L4_{Wi-Fi},\;L5_{Wi-Fi},\;L6_{Wi-Fi},\;L7_{Wi-Fi}\}$ in Figure 5. Similarly, the route can be divided into segments according to the change of the latitude and longitude of GPS data along the segments. The route segment set $L_{lon\&lat}=\{L1_{lon\&lat},\;L2_{lon\&lat},\;L3_{lon\&lat},\;L4_{lon\&lat}\}$ in Figure 6.

**Figure 7:**
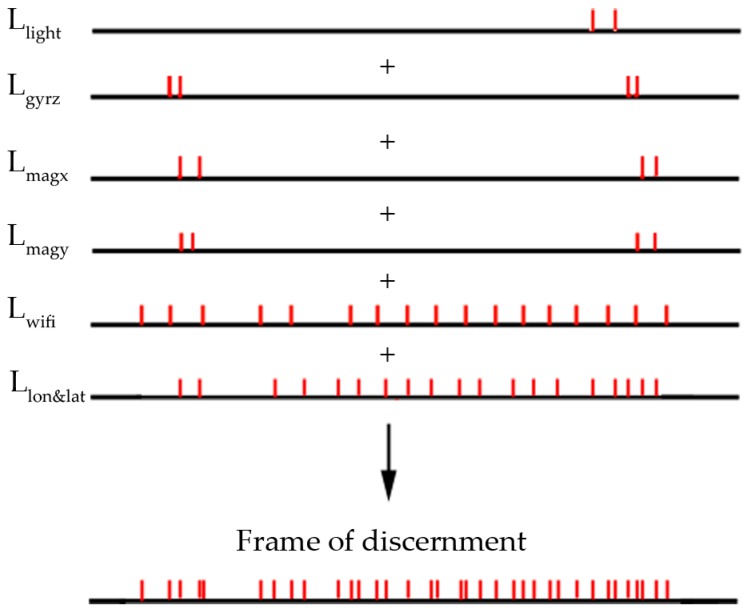
Example of an evidence framework.

This study divides the target route into many tiny segments by intersecting the route segment set of all sensors, which is viewed as the frame of discernment, θ. A schematic diagram of the derived framework is shown in Figure 7. Each tiny route segment has a characteristic combination of sensors, namely, $\theta =\{L_{Magx}\cup L_{Magy}\cup L_{Wi-Fi}\cup L_{Light}\cup L_{Gyro}\cup L_{Lon\&Lat}\}$. In this implementation, θ is represented as:

(4) $$\begin{array}{cc} \theta_{i}=\{ & Magx=\left(Magx_{s},Magx_{ave|s=1},Magx_{\max|s=2},Magx_{\min|s=2},Magx_{slope|s=2}\right),\\ & Magy=\left(Magy_{s},Magy_{ave|s=1},Magy_{\max|s=2},Magy_{\min|s=2},Magy_{slope|s=2}\right),\\ & Gyro=\left(Gyro_{s}\right),\\ & Wi-Fi=\left(Wi-FiMAC_{Num},Wi-FiMAC_{name1},\ldots ,Wi-FiMAC_{name_{Num}},Wi-FiRSSI_{1},‥‥Wi-FiRSSI_{num}\right),\\ & Light=\left(Light_{\max},Light_{\min},Light_{ave}\right),\\ & Lon\&Lat=\left(Lon_{start},Lat_{start},Lon_{end},Lat_{end}\right)\} \end{array}$$

(5) $$\theta =\{\theta_{1},\theta_{2},\ldots ,\theta_{i},\ldots ,\theta_{n}\}$$

where *i* is the serial number of a route segments in the target route, and $\theta_{i}$ is the discernment within route segment *i*. The status of magnetometer data along the x- and y-axis $Magx_{s}=\left\{1,2\right\}$ and $Magy_{s}=\left\{1,2\right\}$ is such that 1 indicates that the data are stable and 2 indicates an obvious change in the data. $Magx_{ave|s=1}$ is the average of the x-component of the magnetometer data when the status is 1. $Magx_{\max|s=2},\;Magx_{\min|s=2}\;\mathrm{and}\;Magx_{slope|s=2}$ are the maximum, minimum, and slope of magnetometer data in the x-orientation. $Magy_{ave|s=1},Magy_{\max|s=2},Magy_{\min|s=2},Magy_{slope|s=2}$ have similar meanings in the y-orientation. $Gyro_{s}$ is the status of walking reflected by the gyroscope data, $Gyro_{s}=\left\{-1,0,1\right\}$, which indicates turning left, walking straight ahead, and turning right, respectively. $Wi-FiMAC_{Num}$ and $Wi-FiRSSI_{Num}$ indicate the number of Wi-Fi MAC addresses and Wi-Fi RSSI respectively along the route segment and $Wi-FiMAC_{name1},\ldots ,Wi-FiMAC_{name_{Num}}$ and $Wi-FiRSSI_{1},\ldots ,Wi-FiRSSI_{Num}$ are the list of them. $Light_{\max},Light_{\min},Light_{ave}$ represent the maximum, minimum, and average light values in this route segment. $Lon_{start},Lat_{start},Lon_{end},Lat_{end}$ are the longitude and latitude of the start and end points of the route segment.

**Figure 8:**
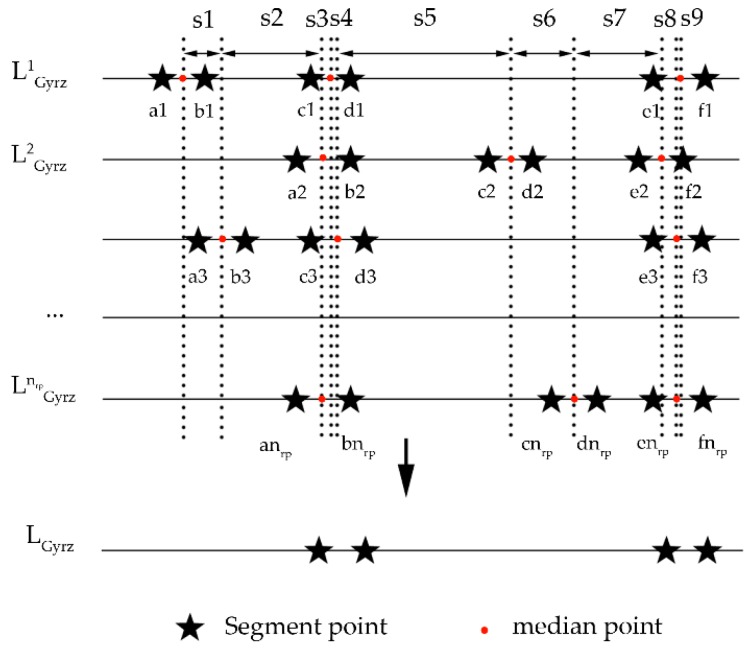
Example of optimized method of gyroscope frameworks’ segmentation.

The determination of the above mentioned evidence framework is based on single data source. To a certain extent, the framework would bring about incorrect segment results due to external interference. For example, when encountering a temporarily parked vehicle in the process of targeting road, the record of magnetometer would fluctuate and generate redundant segment points. In addition, when data collector changes direction in order to avoid pedestrian, the record of gyroscope reaches peak or valley, which also affects the correctness of the framework. In order to eliminate above mentioned interferences, this paper optimizes the framework by using multiple repeated data sources. In this paper, each sensor data were collected $n_{rp}$ times. They were divided as $L_{Magx}^{all}=\left\{L_{Magx}^{1},L_{Magx}^{2},\cdots ,L_{Magx}^{n_{rp}}\right\},\cdots L_{Lon\&Lat}^{all}=\left\{L_{Lon\&Lat}^{1},L_{Lon\&Lat}^{2},\cdots ,L_{Lon\&Lat}^{n_{rp}}\right\}$ according to the above method. Then, this paper found the common segmentations of framework was the optimized framework of sensors. Figure 8 illustrates all frameworks of gyroscope. $L_{Gyro}^{r}$ is the framework segmentation result of gyroscope in the collection of the $r^{th}$ times, and s is the distance between two adjacent median points of a given segment route. Since the given GPS accuracy is about 15 m, if the distance between two points is less than 15 m, the two points are considered as a same position. Hence, this paper uses 15 m as the threshold to remove segment points whose median points are far from other median points. Moreover, the characteristic of the segmented section between each two deleted segment points is assigned as same as their previous section. For example, the characteristic of the deleted section {c2,d2} is a straight walk, which is identical with the one of the sections {b2,c2}. After this common segmentation finding, this paper could get a suitable framework for possible pedestrian navigation situations.

##### 3.3.2.2. Basic Belief Assignment

**Figure 9:**
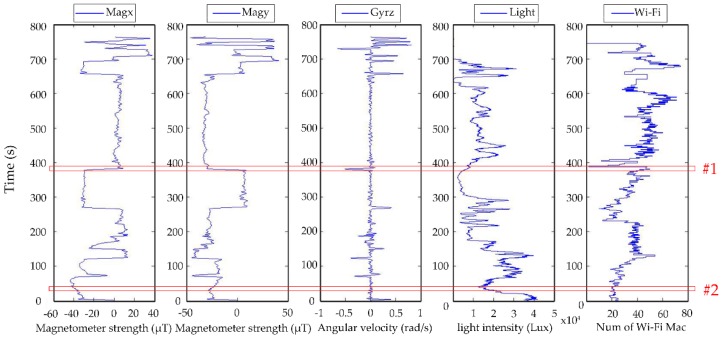
Co-existing relationship of sensor data.

Co-existing relationships of evidence are common phenomena for smartphone sensors used to locate pedestrians as a result of similarities in activity behaviors and constraints in a navigation environment. Usually, the best evidence is when they have co-existing relationships that can reflect the uniqueness of the actual scene in space and time sequences. Therefore, the basic selection rule of co-existing relationships between sensors is that the sensor data feature can be readily detected by simple filtering algorithms, such as moving windows, and the combination of sensors in each route segment or the sensor features presents an obvious difference between the neighboring route segments. For smartphones’ sensors in pedestrian navigation environments, the data from the magnetometer, Wi-Fi, and GPS are independent environmental information, and these data obtained at each route segment could become evidence of a unique environment. The gyroscope mainly detects the changes (or turns) in the journey through the environment so that the gyroscope can be added to the evidence framework when the real-time gyroscope data exhibits changing features. For example, Figure 9 shows an example of the co-existing relationship of sensor signals in a target route. Areas #1 and #2 are two subset locations of the framework. The sensor data characteristics of the two subsets are listed in Table 1. In area #1, the x- and y-components of the magnetometer data show a sudden increases and decreases, respectively; the gyroscope shows features that are consistent with a right turn, and the light data are in keeping with features observed outdoors. Therefore, there is a co-existing relationship between the signals of the x- and y-components of the magnetometer, the gyroscope z-component, and the light-level. Therefore, the values of the four sensors in area #1 can be viewed as an evidence combination for defining the basic belief probability distribution. Similarly, for area #2, the gyroscope does not have a co-existing relationship of evidence with other sensors because the gyroscope exhibits stable behavior. Therefore, the combination of evidence and the probability distribution only include data of sensors such as the magnetometer x- and y-components, light, and Wi-Fi.

After selecting sensors as a co-existing sensor combination for each route segment, our proposed approach calculates the similarity of the real-time data of co-existing sensors with a predefined evidence framework, according to the following Equations (6)–(15):

(6) $$SimMag(Magx,Magx^{\prime })=\{\begin{array}{cc} f\left(Magx_{ave|s=1},Magx_{ave|s=1}^{\prime }\right) & Magx_{s}=Mag{x_{s}}^{\prime }=1\\ \left[\begin{array}{l} f\left(Magx_{Max|s=1},Magx_{Max|s=1}^{\prime }\right)+\\ f\left(Magx_{Min|s=1},Magx_{Min|s=1}^{\prime }\right)+\\ f\left(Magx_{slope|s=1},Magx_{slope|s=1}^{\prime }\right) \end{array}\right]/3 & Magx_{s}=Mag{x_{s}}^{\prime }=2\\ 0 & Magx_{s}\neq Mag{x_{s}}^{\prime } \end{array}$$

(7) $$f(x,x^{\prime })=1-\left|x-x^{\prime }\right|/x$$

(8) $$SimGyro(Gyro_{s},Gyro_{s}^{\prime })=\{\begin{array}{cc} 1 & Gyro_{s}=Gyro_{s}^{\prime }\\ 0 & Gyro_{s}\neq Gyro_{s}^{\prime } \end{array}$$

(9) $$SimWi-Fi\left(Wi-Fi_{s},Wi-Fi_{s}^{\prime }\right)=SimName\left(Wi-Fi_{s},Wi-Fi_{s}^{\prime }\right)\times SimRSSI(WF\cap WF^{\prime })$$

where

(10) $$SimName\left(Wi-Fi_{s},Wi-Fi_{s}^{\prime }\right)=\frac{1}{2}f(Wi-FiMAC_{num},Wi-FiMAC_{num}^{\prime })+\frac{\left|WF\cap WF^{\prime }\right|}{2|WF\cup WF^{\prime }|}$$

(11) $$SimRSSI(WF\cap WF^{\prime })=\frac{w_{RSSI}}{n}\sum_{i\in WF\cap WF^{\prime }}(RSSI_{i}-RSSI_{i^{\prime }})/\left|RSSI_{i}\right|$$

(12) $$\begin{array}{l} WF=\left\{Wi-FiMAC_{name1},\cdots ,Wi-FiMAC_{nameNum}\right\},\\ WF^{\prime }=\left\{Wi-FiMAC_{name1}^{\prime },\cdots ,Wi-FiMAC_{name1}^{\prime }\right\}. \end{array}$$

(13) $$SimLight(Light,Light^{\prime })=\left[\begin{array}{l} f\left(Light_{Min},Light_{Min}^{\prime }\right)+\\ f\left(Light_{Max},Light_{Max}^{\prime }\right)+\\ f\left(Light_{Ave},Light_{Ave}^{\prime }\right) \end{array}\right]/3$$

Here, $w_{RSSI}$ is the weight relatively to weight of Wi-Fi Mac address while matching the sensors with predefined evidence framework. $RSSI_{i}$ and $RSSI_{i^{\prime }}$ are RSSI measurement values of Wi-Fi access point *i* in evidence framework and it in the real-time data set.

The light sensor is more complex than other sensors in that the intensity of light varies greatly across time and climate. The most accurate match result can be obtained when the light condition of Light is similar with the $Light^{\prime }$. Hence, this paper introduces a light similarity method which considers the continuity of data. In this method, the intensities of light under different light conditions are processed according to Section 3.3.2.1. The light segment result can be obtained as $L_{light}=\left\{\left\{L1_{light}^{1},\cdots ,Ln_{1}{}_{light}^{1}\right\},\cdots ,\left\{L1_{light}^{c},\cdots ,Ln_{c}{}_{light}^{c}\right\}\right\}$, c denotes the amount of light condition, such as day, night, sunny day, cloudy day, etc. $Li_{light}^{ci}$ is the ith section when light_condition=ci. Then, we calculate the SimLight of real-time data with $L_{light}$ according to Equation (13) in m times. The number of successful match results in different light conditions is represented as $Num_{i},i\in \left[1,c\right]\cap 0<Num_{i}<m$. If light_condition=ci and $Num_{ci}=Max\left(Num\right)$, we regard the ci as the most similar light condition of real-time data. Finally, the segment route whose light condition is ci is selected.

Table 2 is an example of selection of light segment. $Light_{\Gamma }$ represents the Γth real-time light data. $L_{light}-Light_{\Gamma }$ represents the match result of segment data under different light conditions.

(14) $$L_{light}-Light_{\Gamma }=\left\{ \begin{array}{l} 0\;success\\ 1\;failure \end{array}\right.$$

(15) $$\begin{array}{l} SimLon\&Lat(Lon\&Lat,Lon^{\prime }\&Lat^{\prime })=f(Lon_{start}-Lon_{end},Lon_{start}^{\prime }-Lon_{end})+\\ \;\;\;\;f(Lat_{start}-Lat_{end},Lat_{start}^{\prime }-Lat_{end}) \end{array}$$

where SimMag, SimGyro, SimWi-Fi, SimLight and SimLon&Lat represent the similarity of the magnetometer, gyroscope, Wi-Fi, light, and latitude and longitude. $Magx^{\prime }$, $Gyro^{\prime }$, $Wi-Fi^{\prime }$, $Light^{\prime }$, $Lon\&Lat^{\prime }$ are the real-time data of sensors that need to be checked for matching the location of pedestrians with the route segment of the evidence framework. Therefore, for real-time data of sensors, this approach will generate a similarity matrix as follows:

(16) $$\left(\begin{array}{cccc} SimMagx_{1} & SimMagx_{2} & \ldots & SimMagx_{n}\\ SimMagy_{1} & SimMagy_{2} & \ldots & SimMagy_{n}^{}\\ SimGyro_{1} & SimGyro_{2} & \ldots & SimGyro_{n}\\ SimWi-Fi_{1} & SimWi-Fi_{2} & \ldots & SimWi-Fi_{n}\\ SimLight_{1} & SimLight_{2} & \ldots & SimLight_{n}\\ SimLon\&Lat_{1} & SimLon\&Lat_{2} & \ldots & SimLon\&Lat_{n} \end{array}\right)$$

where n is the number of discernment in the frame of evidence theory.

In order to improve the error tolerance performance of the matching process, this study finds the five most similar discernments in the frame of evidence using Equations (6)–(15), and ranks them with levels of 1–5. Table 3 lists the values of 1–5 and their corresponding evaluation characteristics. The Equation (17) shows an example of a similarity matrix with 6 discernments.

(17) $$\begin{array}{c} SimMagx\\ SimMagy\\ SimGyro\\ SimWi-Fi\\ SimLight\\ SimLon\&Lat \end{array}\left(\begin{array}{cccccc} 1 & 5 & 2 & 3 & & 4\\ 3 & 2 & 1 & 4 & 5 & \\ 5 & 4 & 1 & 2 & & 3\\ & 2 & 3 & 1 & 4 & 5\\ 4 & & 1 & 3 & 5 & 2\\ & 1 & 4 & 5 & 2 & 3 \end{array}\right)\Rightarrow \left(\begin{array}{cccccc} 0.44 & 0.08 & 0.22 & 0.14 & & 0.11\\ 0.14 & 0.22 & 0.44 & 0.11 & 0.08 & 2.28\\ 0.08 & 0.11 & 0.44 & 0.22 & & 0.14\\ & 0.22 & 0.14 & 0.44 & 0.11 & 0.08\\ 0.11 & & 0.44 & 0.14 & 0.08 & 0.22\\ & 0.44 & 0.11 & 0.08 & 0.22 & 0.14 \end{array}\right)$$

The Equation (17) gives an example of similarity matrix with 6 discernments (left part of ⇒) and its probability matrix m(S) (right part of ⇒).

This study uses Equation (18) to calculate the probabilities of the five most similar discernments. The probability is used to build up a basic belief assignment of each sensor for real-time sensor data.

(18) $$m\left(S_{i}\right)=\frac{{\left(V_{i}\right)}^{-1}}{\sum_{i=1}^{i=5}{\left(V_{i}\right)}^{-1}}$$

where $m(S_{i})$ denotes the probability value of the focal element whose similarity level is i. The right part of Equation (17) shows the probability matrix corresponding to the similarity matrix of the left part of Equation (17).

By combining the co-existing rules of evidence, this study proposes an improved theory of evidence combination formula as follows.

(19) $$M\left(A\right)=\left\{ \begin{array}{cc} \frac{1}{K}\sum_{\cap A_{i}=A}\prod_{1\leq i\leq n}m_{i}\left(A_{i}\right) & A\neq \phi ,\;F_{i}\neq 0\\ 0 & A=\phi \end{array}\right.$$

where K is a normalization constant, $m_{i}$ is the BPA of evidence i, and A is the subset of evidence framework. $F_{i}\neq 0$ means that only the evidence i with characteristic changes are used in the basic belief assignment and evidence combination.

##### 3.3.2.3. Conflict of Evidence Processing

In the process of evidence combination, two factors affect the accuracy of sensor data, one is data latency, the other is external interference. Conflicts of evidence may exist in the matching process of sensor data and evidence. Therefore, it is necessary to deal with this issue during evidence combination. Several approaches have been proposed to manage conflicts in D-S evidence theory, such as, averaging [65], and combining conflict evidence [67], and weighting evidence (evidence pretreatment) [68,69]. Assigning a small probability value to the frame of discernment is a practical operation when determining the basic belief assignment. Therefore, this study combines the approaches of Yager combination rules [66] and weighted evidence to handle the conflict of evidence.

**Figure 10:**
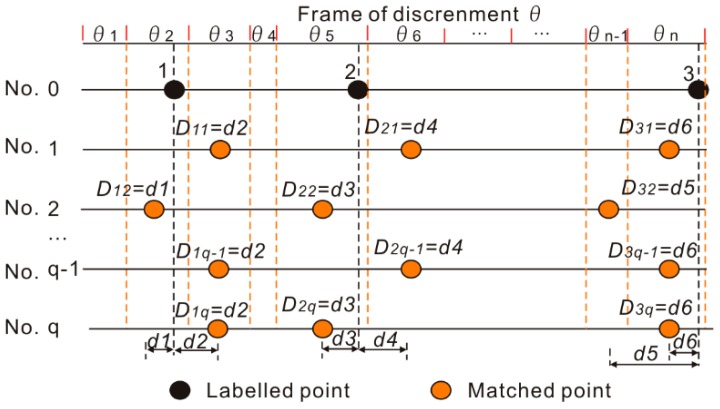
Calculating the matching errors for assigning the weight of sensor *i*.

Weighting evidence is crucial to improving the accuracy of the evidence combination results in the case of different evidence association rules. The weights are usually subjectively assigned, or can be objectively calculated in the presence of historical data sets [70]. In this study, the sensor data sets of the target route are precollected to build the framework of discernment. Hence, they can be preprocessed with objective calculations to obtain the weights for the evidence before the evidence is combined. The detail determining process of sensor’s weight is described as follows:

Figure 10 illustrates an example of calculating the matching errors for assigning weights of sensor *i*. Let Ns indicate the number of sensors, q represents the repeated times of collecting historical data set along the target route, θ is the frame of discernment of the target route, and *u* is the number of labelled points. The matching error can be estimated by comparing the location of the labelled points with the corresponding matched route segments. $L_{j\omega }^{i}$ indicates the matched route segment of sensor *i* at times ω for the corresponding labelled point j. This study assigns the weight of sensor *i* according to a basic rule that if the matching error of a sensor is smaller, the weight of this sensor is higher. Therefore, the weight of sensor *i* is defined as follows:

(20) $$w_{i}=\frac{{\left(\frac{1}{uq}\sum_{j=1}^{u}\sum_{\omega =1}^{q}D_{j\omega }^{i}\right)}^{-1}}{\sum_{i=1}^{Ns}{\left(\frac{1}{uq}\sum_{j=1}^{u}\sum_{\omega =1}^{q}D_{j\omega }^{i}\right)}^{-1}}$$

(21) $$D_{j\omega }^{i}=\left|Loc_{j}-(\theta_{start}^{L_{j\varpi }^{i}}+\theta_{end}^{L_{j\varpi }^{i}})/2\right|$$

where $Loc_{j}$ is the location of labelled point j in θ, and $\theta_{start}^{L_{j\varpi }^{i}},\theta_{end}^{L_{j\varpi }^{i}}$ represent the start and end locations of $L_{j\omega }^{i}$. For example, in Figure 10, the calculated distances between the matched points and labelled point 1 are *D*_11_, *D*_11_, *D*_1*q*-1_, *D*_1*q*_. Here, $\frac{1}{uq}\sum_{j=1}^{u}\sum_{\omega =1}^{q}D_{j\omega }^{i}$ = [(*D*_11_ + *D*_11_ + …… + *D*_1*q*-1_ + *D*_1*q*_) + (*D*_21_ + *D*_21_ + …… + *D*_2*q*-1_ + *D*_2*q*_) + (*D*_31_ + *D*_31_ + …… + *D*_3*q*-1_ + *D*_3*q*_)]/3*q* in the case of sensor *i*.

Then, this study improves the theoretical formula for evidence by employing two strategies. The first is to assign a small probability to the whole frame θ, which makes the inconsistency of evidence negligible [67]. The other strategy is to add weights of the sensors belonging to the evidence, which reflect the accompanying influence of sensors on estimated results. Therefore, the improved theory of evidence combination formulas can be further defined as

(22) $$M\left(A\right)=\left\{ \begin{array}{cc} \frac{1}{K}\sum_{\cap A_{i}=A}\prod_{1\leq i\leq n}m_{i}\left(A_{i}\right)\cdot w_{i}=\frac{1}{K}\sum_{\cap A_{i}=A}\prod_{1\leq i\leq n}m_{i}\left(A_{i}\right)\frac{{\left(\frac{1}{uq}\sum_{j=1}^{u}\sum_{\omega =1}^{q}D_{j\omega }^{i}\right)}^{-1}}{\sum_{i=1}^{Ns}{\left(\frac{1}{uq}\sum_{j=1}^{u}\sum_{\omega =1}^{q}D_{j\omega }^{i}\right)}^{-1}} & A\neq \phi ,\;F_{i}\neq 0\\ 0 & A=\phi \end{array}\right.$$

where $w_{i}$ denotes the weight of evidence i.

## 4. Experiments

This section introduces the experimental environment and collected data for a target route. Then, it gives the calculated frame of evidence of the improved approach, which is similar as a system training. Finally, this section demonstrates the advantages of the proposed approach by comparing the matched results with those obtained by the traditional D-S theory, GPS locations outdoors, and Wi-Fi locations indoors.

### 4.1. Experimental Environment and Data

**Figure 11:**
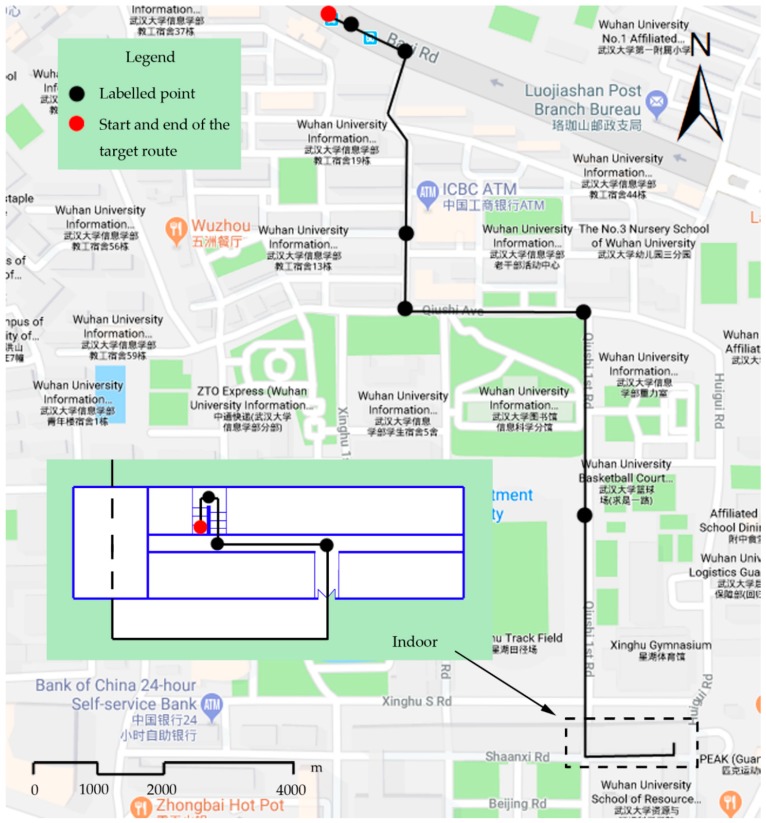
Experimental route and markers.

The experiment was conducted on the campus of Wuhan University and a business card route for pedestrians from the entrance of Wuhan University to the No. 2 School Building was selected (see Figure 11). This study collected the sensor data of the route on 10 occasions by a data acquisition application written by the authors and run in the Android operating system of a VIVI X6 smartphone. The first instance was used to build up the frame for discernment, while the data collected on the other nine times were used to calculate the weights of sensors in the proposed approach. In addition, this study selected nine labelled points to check the matching error of the proposed approach. The nine labelled points are waypoints along the trajectory. Of them, six points were located outdoors and the other three were indoors. During the data acquisition process, sensor data were sampled at a frequency of 20 Hz, and the collected data were stored as a separate .txt file for each repetition. The collected files were processed in MATLAB.

The data format collected in this study is listed in Table 4:

Here, RSSI is received signal strength indicator, which is a measurement of how well your device can hear a signal from an access point or router. Due to the situation that RSSI have large fluctuation over the same distance and are attenuated, this study used the radio propagation model [71] to estimate the relationship between the signal strength indicator and distance. To reduce the influence of RSSI measurement on the evidence framework, this study set a relatively low weight (e.g., $w_{RSSI}$ = 1/10) to RSSI values of the evidence of Wi Fi MAC.

### 4.2. Implementation of the Proposed Approach

**Figure 12:**
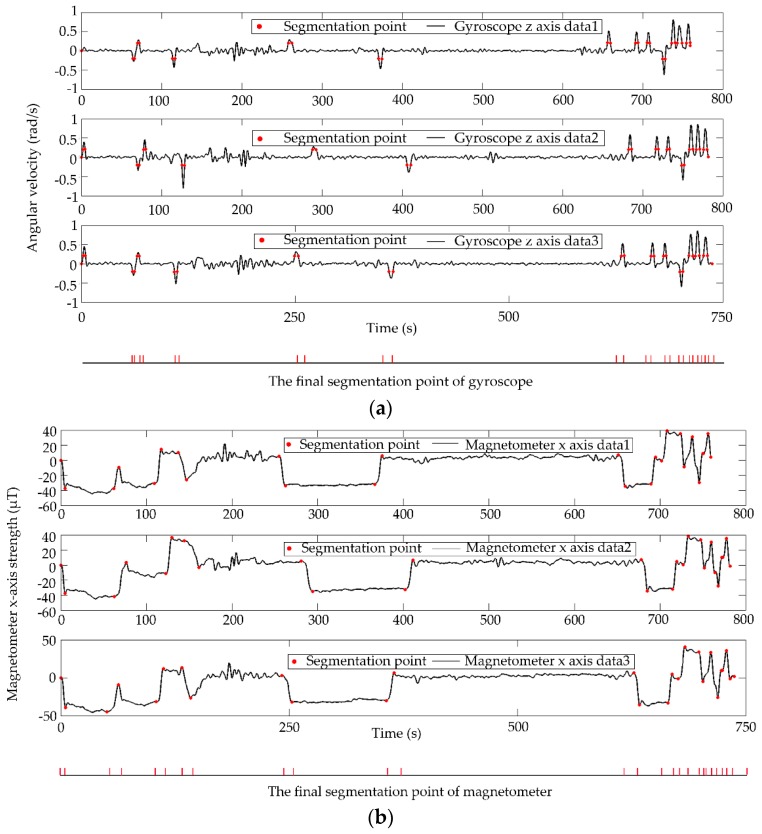

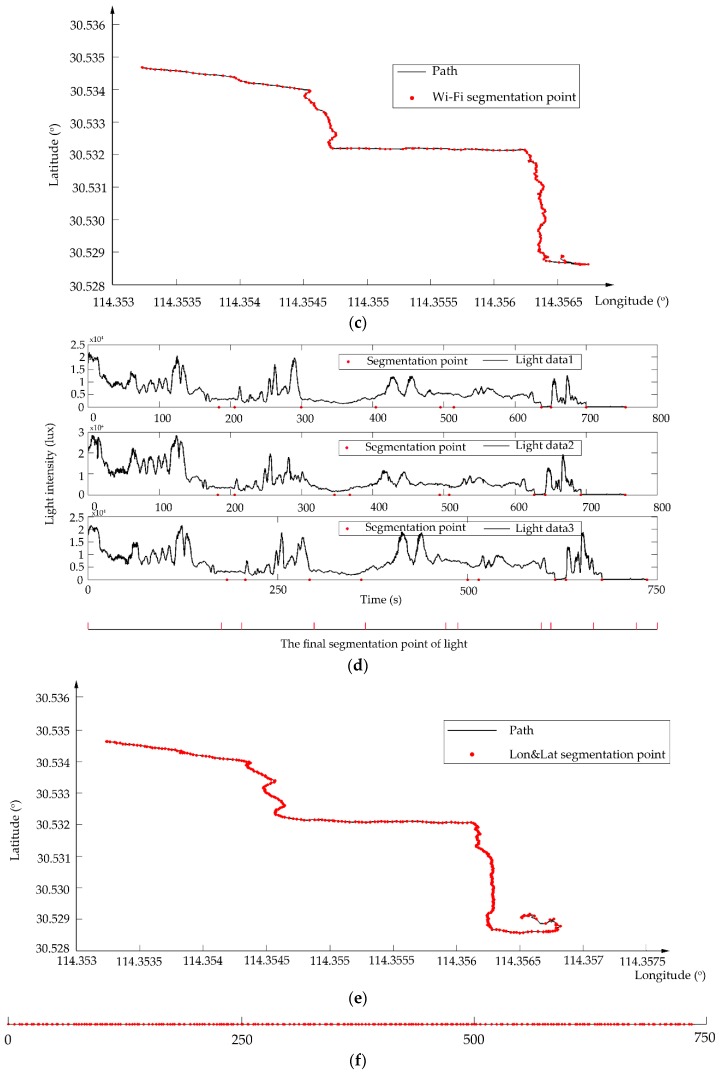
Sensor segmentation results and frame of discernment: (**a**) gyroscope; (**b**) magnetometer; (**c**) Wi-Fi; (**d**) light; (**e**) longitude and latitude; (**f**) frame of discernment.

Figure 12 illustrates the data of the target route from the first collection. The red points in Figure 12a–e represent the segmentation points of sensor data. Figure 12f shows the constructed frame of discernment for the proposed approach.

In addition, five pieces of data in daytime and the same numbers in nighttime are selected to examine the accuracy of the proposed light condition selection method based on the continuity of light. In Table 5, Day time and Night time are two kinds of light conditions defined in our framework. As shown in Table 5, the accuracy achieves 100%.

Based on the determined framework, this study calculated the basic belief assignment of each sensor in the frame of discernment shown in Table 6. In this table, $A_{1},\ldots ,A_{n}$ are subsets of the evidence framework θ. The basic belief assignments of each sensor for the sub-proposition $m\left\{A_{n}\right\}$ are listed, which is calculated according to the five most similar sensor data. According to the approach of Murphy [67] for combining belief functions when conflicts of evidence occur, setting m{θ}=0.01 helps to avoid failure or error caused by conflicts of evidence. The initial probabilities of the five most similar sensors were defined to be 0.44, 0.22, 0.14, 0.11, and 0.01, which are explained in the methodology (see Section 3.3.2.2).

After using the repeated data from collections 2 to 9, this methodology can be used to calculate the distribution of weights of co-existing evidence by the rule that a sensor with a smaller average error has a greater weight in the methodology. Because of the repeatable characteristics of sensors, such as the magnetometer, Wi-Fi, light and GPS along the target route, and two clear conditions of changing and unchanging characteristics of the gyroscope, this study classifies the co-existing situations of sensors into two class, namely, the co-existing situations with and without gyroscope. Table 7 gives the calculated weights for each sensor in the two co-existing situations by using repeated data. GPS is not used in the segments where the GPS signal does not change during a period of time. In the study, the default time threshold is 4 s, which is flexible and can be customized by users.

### 4.3. Experimental Results and Comparative Analysis

To estimate the performance of the proposed approach in terms of match success rate and positioning error of results, this study selected nine (black) labelled points along the target route shown in Figure 11 and recorded the actual locations of them manually when collecting the sensor data.

#### 4.3.1. Comparison of Match Success Rate

The match success rate is the ratio of the success match cases among all experimental cases. Here, the match success rate is calculated as:

(23) $$MSR=\frac{\sum_{i=2}^{10}MS_{i}}{n},MS_{i}=\{\begin{array}{cc} 1 & success\\ 0 & failure \end{array}$$

where MSR is the match success rate, and $MS_{i}$ indicates the match status of the element *i*.

Table 8 gives the match success rate of the nine labelled points along the target route. In this table, #1–#9 indicates the labelled points, ①–⑩ are the serial numbers for data collection, each one of which is a complete collection of sensor data from the origin point to the end point of the target route. The 0 or 1 in the table denotes the match success status by the corresponding approaches, where 0 represents a match failure and 1 represents a successful match.

From Table 8, this study found that the proposed approach could achieve the 100% match success rate for all nine of the labelled points, while the average match success rate of the traditional D-S theory is about 40%. This finding demonstrates the performance of judgment in the case of conflicts of evidence and verifies the feasibility of the proposed method in this study. The proposed method in our paper only use the characteristics of the invisible landmarks to locate pedestrians. Therefore, it can against changes of speed and the time elapsed between invisible landmarks. No matter the distance of two gyroscope events is short or long, the framework section in intermediate time always regarded as the straight section. This method considers it is still between the two invisible landmarks.

#### 4.3.2. Positioning Error of Results

To compare the location error of results, this study uses multiple sensors and GPS when outdoors and multiple sensors and Wi-Fi when indoors. The positioning errors of these approaches, which are the difference between the matched route segments and the actual locations, were evaluated using the nine labelled points. Table 9 and Table 10 present positioning errors in the outdoor (see Figure 11) and indoor environments, respectively. The average positioning error of the proposed approach is less than 5 m outdoors, and less than 3 m indoors, which can meet the positioning needs of pedestrian navigation. Furthermore, the mean positioning errors are smaller than those using GPS or Wi-Fi alone. To estimate the reduced extent of the proposed approach, this study defines a concept of reduced percentage (RP) of positioning errors by the proposed approach when comparing with GPS as follows:

(24) $$RP=\frac{E_{gps}-E_{pDS}}{E_{gps}}\times 100\%$$

where $E_{gps}$ and $E_{pDS}$ denote the positioning errors of the GPS and the proposed D-S approach. The reduced percentages for the six labelled points outdoors are between 15.9% and 54.4% (see Table 9). The equivalents for the three labelled points indoors are 10.1% to 62.6%. Clearly, the results for both the indoor and outdoor environments demonstrate that they offer improved positioning accuracy.

**Figure 13:**
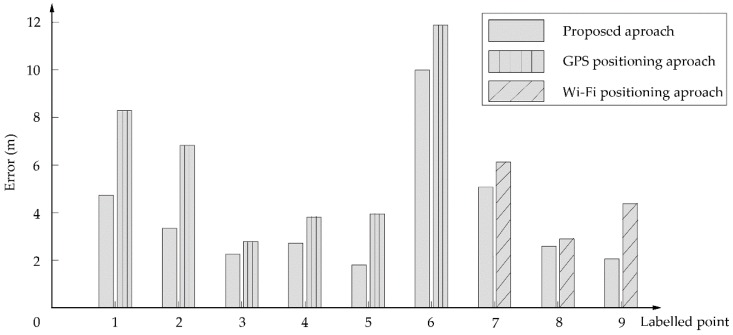
Positioning errors of labelled points.

Figure 13 illustrates the average positioning errors of all nine labelled points obtained by the proposed approach, GPS and Wi-Fi. Among them, the positioning accuracy at the turning position is better than other positions, which may be because sensors, such as gyroscopes and magnetometers, have obvious characteristics when turning. Furthermore, the synthesized results of the proposed approach are obviously better than those derived from GPS or Wi-Fi alone. However, there is a large positioning error for point #6, probably because the path along point #6 is severely blocked by trees, and the weight of GPS is higher than other sensor in the outdoor environment. Furthermore, the intersection area around point #6 is large, and the gyroscopes and magnetometers do not have obvious changing characteristics. This indicates the need to reduce positioning error through the use of more sensitive evidence.

## 5. Conclusions

In this study, the changing characteristics and combinations of various sensors’ data in smartphones or navigation devices are viewed as invisible salient landmarks for predefined business card route of pedestrian navigation. This study introduces an improved Dempster-Shafer theory of evidence to find invisible landmarks along predesigned pedestrian routes without using digital maps by integrating the co-existing phenomenon of sensors’ signal change characteristics. This approach distinguishes navigation methods from fingerprint-based localization and navigation applications by focusing on combinational features of signal changes, rather than on individual signal changes. Moreover, it integrates a proposed similarity measure of real-time data of co-existing sensors with a predefined evidence framework for refining the basic belief assignment in the theory of evidence, and a match error-based sensor weight assignment approach for handling the conflict of evidence processing. Furthermore, this paper optimizes the framework for possible pedestrian navigation situations by using multiple repeated data sources to eliminate temporally incorrect segment results, e.g., the external interference when encountering a parked vehicle or avoiding pedestrians. In this approach, the frame of discernment is utilized according to the path division by sensors’ characteristics. The real-time pedestrian’s sensor data features are extracted to contrast and match with the framework. As result, this study improves the evidence theory to fuse the matching results of each sensor and infer a pedestrian’s location. The proposed approach is tested in the real experiment environment of pedestrian navigation. The experimental results shows the proposed approach could achieve the 100% match success rate for all labelled points, while the average match success rate of the traditional D-S theory is about 40%. This approach also improved the positioning accuracy of 15.9% and 54.4% for labelled points outdoors and 10.1% to 62.6% for labelled points indoors when it is compared with GPS or Wi-Fi alone in the study area. These experiments demonstrate that the proposed approach outperforms those based on GPS or Wi-Fi alone in the study area. Also, this approach can be applied seamlessly both indoors and outdoors for newcomers to follow predesigned business card routes.

There is scope for further research following this proposed approach. The first avenue it to combine the mapless pedestrian navigation with digital maps, which aims to make full use of visual and invisible landmarks within pedestrian navigation environments. The second avenue is to improve positioning sensors or create an adaptive smartphone-dependent sensor weight assignment method. The third avenue is to produce invisible landmark-based navigation maps and integrate these invisible landmarks into current landmarks or point-of-interest-based pedestrian navigation data models. The fourth avenue is to create comparative navigation in future work.

## Figures and Tables

**Table 1:** An example of the co-existing relationships of sensors in a subset of framework.

	Mag x	Mag y	Gyr z	Light	Wi-Fi
#1	Increase	Decrease	Right turn	Stabilization	47
	√	√	√	√	√
#2	Stable	Stable	Stable	Fluctuation	20
	√	√		√	√

**Table 2:** Example of selecting light segment result.

Light Condition	$L_{light}-Light_{1}$	$L_{light}-Light_{2}$	$L_{light}-Light_{\Gamma }$	$L_{light}-Light_{m}$	Num	Max (Num)
1	0	0		0	0	/
2	0	1		0	m-2	/
						/
C	1	0		1	m-1	√

**Table 3:** Level of similarity and their evaluating characteristics.

Values	Evaluating Characteristics
1	Extremely high
2	High
3	Medium
4	Low
5	Relatively low

**Table 4:** Example of experimental data format.

**Timestamp**	**Longitude**	**Latitude**	**Gyroscope z-axis**	**Light**
20171203135331900	114.XXXXXXX	30.XXXXXXX	0.013504496	24,475
20171203135332100	114.XXXXXXX	30.XXXXXXX	0.013215541	24,182
**Magnetometer x-axis**	**Magnetometer y-axis**	**Wi Fi MAC**	**Wi Fi Name**	**Wi Fi RSSI**
−34.5	−22.859	e0:4f:bd:80:09:69	ChinaNet-3upP	−73
−34.5	−23.1	e0:4f:bd:80:09:69	ChinaNet-3upP	−73

**Table 5:** Selection of light segmentation based on continuity of data matching.

Real-Time Light Condition	Day Time	Night Time	Accuracy
Day time	5	0	100%
Night time	0	5	100%

**Table 6:** Basic belief assignment of each sensor in the frame of discernment.

	Mag x	Mag y	Gyro z	Wi-Fi	Light	GPS
$m\left\{A_{1}\right\}$	0.44	0.08	0	0.08	0.12	0.44
$m\left\{A_{2}\right\}$	0.22	0.44	0.08	0.44	0	0.11
$m\left\{A_{3}\right\}$	0.14	0.22	0.44	0	0.44	0.08
$m\left\{A_{4}\right\}$	0.11	0.14	0.22	0.14	0.22	0.14
$m\left\{A_{5}\right\}$	0.08	0.11	0.11	0.11	0.14	0
$m\left\{A_{n}\right\}$	0	0	0.14	0.22	0.08	0.22
m{θ}	0.01	0.01	0.01	0.01	0.01	0.01

**Table 7:** Weight of evidence.

	Situations	Mag x	Mag y	Gyr z	Wi-Fi	Light	GPS
Outdoor	With gyroscope	0.003	0.004	0.205	0.143	0.005	0.64
	Without gyroscope	0.007	0.012	0	0.397	0.016	0.568
Indoor	With gyroscope	0.01	0.01	0.75	0.223	0.007	0
	Without gyroscope	0.02	0.034	0	0.92	0.026	0

**Table 8:** Match success rate of the proposed approach and traditional D-S theory.

Labelled Point	Approach	Serial Number of Data Collecting										Match Success Rate
		①	②	③	④	⑤	⑥	⑦	⑧	⑨	⑩	
#1	Proposed approach	1	1	1	1	1	1	1	1	1	1	100%
	Traditional D-S theory	1	0	1	0	0	1	0	1	0	1	50%
#2	Proposed approach	1	1	1	1	1	1	1	1	1	1	100%
	Traditional D-S theory	1	0	0	0	0	0	0	0	0	0	10%
#3	Proposed approach	1	1	1	1	1	1	1	1	1	1	100%
	Traditional D-S theory	1	0	0	0	0	0	0	0	0	0	10%
#4	Proposed approach	1	1	1	1	1	1	1	1	1	1	100%
	Traditional D-S theory	1	0	0	0	0	0	0	0	0	0	10%
#5	Proposed approach	1	1	1	1	1	1	1	1	1	1	100%
	Traditional D-S theory	1	0	0	0	0	0	0	0	0	0	10%
#6	Proposed approach	1	1	1	1	1	1	1	1	1	1	100%
	Traditional D-S theory	1	1	1	1	1	1	1	1	1	0	90%
#7	Proposed approach	1	1	1	1	1	1	1	1	1	1	100%
	Traditional D-S theory	1	0	1	1	0	1	0	1	1	0	60%
#8	Proposed approach	1	1	1	1	1	1	1	1	1	1	100%
	Traditional D-S theory	1	0	0	1	1	1	0	1	0	1	60%
#9	Proposed approach	1	1	1	1	1	1	1	1	1	1	100%
	Traditional D-S theory	1	0	0	0	1	1	0	1	1	1	60%

**Table 9:** Positioning errors (in meters) of the proposed approach (abbreviated as “Our” in the table) and GPS in outdoor environments.

Labelled Point	Locating Method	Serial Number of Data Collecting										Mean Error (m)	RP (%)
		①	②	③	④	⑤	⑥	⑦	⑧	⑨	⑩		
#1	Our	0.21	5.94	2.22	8.34	10.71	1.68	1.68	2.43	10.71	3.39	4.73	42.9
	GPS	0.57	24.15	2.21	14.55	14.55	2.21	4.24	3.94	14.55	1.91	8.29	
#2	Our	0.18	2.79	1.83	4.98	6.21	8.7	3.15	1.83	1.83	1.83	3.33	51.2
	GPS	0.51	8.66	4.58	10.73	10.73	10.73	8.66	4.58	4.58	4.58	6.83	
#3	Our	0.51	2.34	6.6	3.51	1.11	3.51	1.77	1.26	0.57	1.35	2.25	19.1
	GPS	0.51	0.64	8.55	3.79	1.01	3.79	3.79	3.04	1.01	1.69	2.78	
#4	Our	0.48	2.73	4.92	3.30	1.65	1.77	3.3	4.23	2.91	1.86	2.71	28.7
	GPS	0.51	0.64	6.38	5.25	5.25	0.64	8.03	4.99	3.64	2.25	3.80	
#5	Our	0.51	1.83	2.28	0.93	2.19	2.28	1.23	2.28	2.19	2.28	1.80	54.4
	GPS	0.51	4.31	2.93	1.16	2.93	4.31	14.59	2.93	2.93	2.93	3.95	
#6	Our	0.54	5.37	15.27	10.5	14.07	10.5	12.39	14.73	7.14	9.45	9.99	15.9
	GPS	0.54	6.30	17.21	11.81	13.13	13.13	15.86	18.56	10.43	11.81	11.88	

**Table 10:** Positioning errors (in meters) of the proposed approach (abbreviated as “Our” in the table) and Wi-Fi in indoor environments.

Labelled Point	Locating Method	Serial Number of Data Collecting										Mean Error (m)	RP (%)
		①	②	③	④	⑤	⑥	⑦	⑧	⑨	⑩		
#7	Our	0.15	3.00	2.40	3.00	3.00	3.00	3.00	0.78	16.74	14.91	4.99	62.6
	Wi-Fi	0.99	2.75	12.475	5.875	2.75	2.75	2.75	0.675	17.70	12.45	6.12	
#8	Our	0.39	2.19	5.91	1.5	7.98	1.35	2.94	1.5	1.5	0.63	2.59	10.1
	Wi-Fi	0.63	1.375	1.925	3.075	3.075	1.375	3.625	3.625	6.475	3.625	2.88	
#9	Our	0.21	1.77	3.57	3.03	3.03	1.62	3.03	0.6	3.03	0.6	2.05	53.2
	Wi-Fi	0.6	1.225	1.225	4.40	5.375	1.5	9.875	3.3	1.225	14.975	4.38	
